# Vibrotactile augmentation enhances late-phase control in sequential reaching without accuracy costs

**DOI:** 10.3389/fnhum.2026.1794200

**Published:** 2026-04-24

**Authors:** Saba Mohammadalinezhad Kolahdouz, Quinn Malone, Steven R. Passmore, Jonathan J. Marotta, Cheryl M. Glazebrook

**Affiliations:** 1 Perceptual Motor Integration Lab, Applied Health Sciences, Faculty of Kinesiology and Recreation Management, University of Manitoba, Winnipeg, MB, Canada; 2 Sensorimotor Physiology and Integrative Neuromechanics, Faculty of Health and Social Development, School of Health and Exercise Sciences, University of British Columbia, Okanagan, Kelowna, BC, Canada; 3 Perceptual Motor Behavior Lab, Faculty of Kinesiology and Recreation Management, University of Manitoba, Winnipeg, MB, Canada; 4 Perception and Action Lab, Psychology, Faculty of Arts, University of Manitoba, Winnipeg, MB, Canada

**Keywords:** auditory stimulation, coordination, kinematics, motor activity, sensory integration, vibration

## Abstract

**Introduction:**

Sequential goal-directed movements require the integration of advance planning and online control processes. However, the extent to which augmented sensory feedback influences these processes across multiple movement segments remains unclear. Vibrotactile and auditory feedback may enhance motor control by providing additional sensory information during movement execution, particularly in complex sequential tasks.

**Methods:**

Twenty-four neurotypical adults (mean age 27.04 ± 5.31 years) performed one-target and four two-target reaching tasks involving single- and bimanual movements with extension and reversal components. Participants completed the tasks under three sensory conditions: no feedback, auditory feedback, and vibrotactile feedback delivered at first target contact. Reaction time, movement time, spatial accuracy, and kinematic measures including peak velocity and time after peak velocity were analyzed using repeated-measures analyses of variance.

**Results:**

Reaction time was significantly influenced by both sensory condition and target task complexity, with faster movement initiation observed in the no-feedback condition and during less complex movement sequences. Sensory condition did not significantly affect overall movement time or spatial accuracy. However, vibrotactile feedback significantly reduced time after peak velocity during the second movement segment, indicating enhanced late-phase control without compromising accuracy. Target task complexity also significantly modulated kinematic control, particularly during bimanual reversal movements.

**Discussion:**

These findings demonstrate that vibrotactile feedback selectively enhances deceleration-related control during sequential reaching movements, supporting its role as an effective sensory augmentation strategy for improving movement efficiency in complex motor tasks. Such low-cost sensory interventions may have practical implications for enhancing motor performance in populations experiencing age-related or neurological declines in sensorimotor function.

## 1 Introduction

Goal-directed actions often involve sequential movements toward multiple targets. A central question in the study of sequential actions is how planning a subsequent movement influences the execution of the first. The One Target Advantage (OTA) refers to the finding of faster target acquisition when reaching to a target in isolation compared to reaching to the same target when immediately followed by a second movement. In other words, executing a single reaching movement is typically faster than executing the first movement of a planned two-step movement sequence. This effect has been demonstrated in numerous studies (e.g., Adam et al., 2000; Khan et al., 2010; Lawrence et al., 2013, 2016) with neurotypical adults and adults with Down Syndrome (Lawrence et al., 2013). Evidence suggests that the OTA persists across different effectors and task, including variations in hand use, practice, and visual feedback availability (Adam et al., 2000; Khan et al., 2010; Lavrysen et al., 2002).

Two main theoretical accounts have been proposed to explain the OTA (Adam et al., 2000; Helsen et al., 2001; Lavrysen et al., 2002; Khan et al., 2010; Lawrence et al., 2013, 2016; Bested et al., 2018; Hoffmann, 2017). The movement integration hypothesis proposes that planning for the second movement segment begins before the first movement is completed, causing an overlap in control and planning processes, thus a slowing of the first movement. In contrast, the movement constraint hypothesis posits that the first movement is deliberately slowed or controlled to ensure spatial accuracy and a favorable biomechanical state for the second movement. In this view, performers constrain the initial movement (i.e., end it with more care) so that they can more reliably execute the subsequent action. Task factors like difficulty, movement direction changes, and limb coordination demands can influence which mechanism dominates. For example, high accuracy demands or difficult second targets tend to support the constraint hypothesis (i.e., people sacrifice speed on the first movement to maintain accuracy), whereas easier tasks often show more evidence of overlapping planning, which is more consistent with the integration hypothesis. The two explanations can also coexist. Recent evidence demonstrates that both processes can operate in parallel depending on context with the relative contribution of each task structure, movement complexity, and biomechanical constraints. For instance, Lavrysen et al. (2002) demonstrated that the OTA diminishes or even disappears when the second movement reverses back toward the start (as opposed to continuing in the same direction). In such reversal sequences, participants did not show the typical slowing on the first movement, suggesting that different planning strategies (perhaps more sequential or segmented planning) were at play when the second target required a reversal Lawrence et al. (2013, 2016). Similarly, the OTA persists even when sequential movements are executed bimanually (using two different effectors) (Khan et al., 2010). That is, when the first movement is with one hand and the second with the other. In those cases, the first movement still tends to be slower if a second movement will follow, implying that the interference or planning cost arises from central processing rather than from limb-specific muscular factors. This pattern of results was shown by both Khan et al. (2010) and Lawrence et al. (2013), who found that whether one or two hands are involved, the first segment of a two-target action is generally slower than a single-target movement, pointing to a central (cognitive or neural) origin of the effect.

Given the robust and general nature of the one-target advantage, a question of both theoretical and practical interest is whether this planning-related cost can be modified by external factors. For example, if augmented sensory feedback is provided during the task performance. Enhanced sensory cues (beyond intrinsic vision and proprioception) have been shown to improve motor performance in various contexts by facilitating real-time movement adjustments and reducing reliance on vision or body position (Yazdani, Aghdasi et al., 2020). In particular, auditory and vibrotactile feedback have emerged as useful supplements in motor learning and rehabilitation (Moinuddin et al., 2021; Sigrist et al., 2013). Recent reviews also highlight the rapid growth in home-based digital rehabilitation and wearable feedback systems, supporting the clinical feasibility of low-cost sensory augmentation (Abdulrabba et al., 2025; Rayes et al., 2023; Chan et al., 2026). Moreover, research has found that providing immediate sounds or vibrations related to the movement can heighten a performer’s awareness of movement errors or endpoints, leading to more precise and faster corrections (Bark et al., 2014; Sigrist et al., 2013). For example, congruent visual and vibrotactile cues during a movement task have been found to improve movement quality (e.g., reduced endpoint variability, smoother velocity profiles, and shorter deceleration phases). In addition, vibrotactile-only cues can elicit faster response latencies than visual-only cues in time-critical response paradigms (e.g., faster braking responses to tactile versus visual warning signals), suggesting that tactile channels can support rapid detection and action when visual attention is occupied (Lylykangas et al., 2016).

Because vibrotactile signals are transmitted via fast-conducting Aβ afferents, somatosensory cortical responses can emerge within approximately 20–30 ms following stimulus onset (Allison et al., 1991), allowing rapid integration into ongoing motor commands. Shah et al. (2023) demonstrated that supplemental vibrotactile feedback improved the accuracy and efficiency of planar goal-directed reaching, indicating that vibrotactile cues can significantly reduce endpoint error when reaching without vision. Augmented auditory feedback (e.g., beeps on target hits or deviations) has likewise been shown to aid motor control by providing an immediate external signal that the nervous system can use to adjust ongoing movements in real time (Yazdani, Mohammadalinezhad et al., 2020). Although early auditory brainstem responses occur rapidly, task-relevant cortical auditory processing typically unfolds over longer time windows (~50–100 ms; Picton et al., 2003; Elliott et al., 2010; Faul et al., 2007), which may influence how quickly such feedback can be incorporated for movement correction.

Based on the evidence discussed above, both forms of augmented feedback appear to enhance movement control to some extent. However, whether such feedback can specifically influence sequential movement planning costs, such as those reflected in the one-target advantage (OTA), remains largely unexplored. One recent line of work has begun to examine how augmented sensory feedback interacts with sequential planning demands in older adults. Kolahdouz et al. (2025) reported that vibrotactile feedback reduced reaction time specifically in the two-target single-hand extension condition (2T1He) and was associated with increased peak velocity and shortened time after peak velocity in the second movement segment. A related study including both younger and older adults reported that the specific target task interacted with the sensory condition, with shorter reaction times observed in two-target trials under vibrotactile feedback compared to no feedback (Mohammadalinezhad Kolahdouz et al., 2026). Together these findings suggest that vibrotactile cues may modulate preparatory and late-phase control processes in sequential actions under certain task demands, although the extent to which such effects reflect changes in advance planning costs remains unclear. In the present study, we examined how auditory and vibrotactile feedback affect sequential reaching performance in younger adults when performing extension, reversal and multi-limb movements. Participants performed both simple one-target movements and more complex two-target sequences under three feedback conditions: no augmented feedback, auditory feedback, and vibrotactile feedback. The two-target sequences varied in complexity, including cases where the same hand made two movements in either an extension or a reversal movement, and cases where the two hands each made one movement (bimanual sequences). Based on prior findings, we hypothesized that vibrotactile feedback would provide the greatest benefit to movement performance, particularly for the more complex tasks. Specifically, we expected vibrotactile cues to lead to faster movement initiation and execution in two-target tasks, thereby diminishing the OTA by providing immediate, limb-specific information that enhances planning and control during movement deceleration. We also anticipated that any feedback-related improvements would be more pronounced in the most complex sequences (e.g., bimanual or reversal tasks), where the planning demands and potential for error are highest. By focusing on healthy younger adults (who typically exhibit robust OTA effects under typical aiming conditions), this study aimed to understand if and how added sensory input interacts with motor planning and control processes in the execution of sequential aiming tasks with varying complexity.

## 2 Materials and methods

### 2.1 Participants

An *a priori* power analysis was conducted using G*Power 3.1.9.7 (Faul et al., 2007) to determine the required sample size for the present repeated-measures design. The analysis was informed by effect sizes reported in our prior sequential aiming studies using the One-Target Advantage (OTA) paradigm, which demonstrated medium-to-large within-subject effects for reaction time and kinematic variables (ηp^2^ range ~ 0.15–0.30 across key temporal and spatial measures; Mohammadalinezhad Kolahdouz et al., 2026). Using a repeated-measures ANOVA (within factors) framework with *α* = 0.05, desired power (1- *β*) = 0.80, and a conservative medium effect size estimate (Cohen’s *f* = 0.25), the required sample size was estimated to range between 18 and 22 participants, depending on the assumed correlation among repeated measures. To ensure adequate power across multiple dependent variables and to account for potential data exclusions, we recruited 24 neurotypical right-handed younger adults.

Twenty-four right-handed neurotypical younger adults (14 females; 10 males; 27.04 ± 5.31 years old) participated in the experiment. Right-handedness was an inclusion criterion, as determined by self-report using the Ediburgh Handedness Questionnaire, to control for potential variability in motor planning and execution associated with handedness and hemispheric dominance.

All participants had normal or corrected-to-normal vision and hearing, no recent upper limb injuries, and no history of neurological or musculoskeletal disorders that might affect movement. Participants were recruited through advertisements posted at the University of Manitoba and through community outreach in the surrounding area. The majority of participants were university students, while a smaller proportion consisted of community volunteers not affiliated with the university. Written informed consent was obtained prior to participation. The study protocol was approved by the University of Manitoba Research Ethics Board (protocol #HE2024-0286) and was conducted in accordance with the Declaration of Helsinki (2013 revision).

### 2.2 Apparatus and task setup

Participants performed reaching movements using a handheld stylus outfitted with an infrared-emitting diode (IRED) for three-dimensional motion tracking. The stylus used in this experiment was a commercially available capacitive stylus (Penyeah 4-in-1 Stylus Pen; length = 14.2 cm; diameter = 0.9 cm; base weight = 21 g). For motion capture, a single infrared-emitting diode (IRED) marker was affixed 1.2 cm from the distal end of the stylus. The IRED was positioned on the right lateral side of the stylus to ensure unobstructed optical tracking during grasp.

In the vibrotactile feedback condition, a brushless DC vibration motor was mounted 2.9 cm proximal to the stylus tip (measured from the contact surface of the disc tip). The vibration motor was positioned on the left lateral side of the stylus, opposite the IRED, to minimize interference and maintain balanced grip ergonomics. The separation of the IRED and motor across opposite lateral surfaces reduced mechanical vibration artifacts in the optical signal and ensured stable marker tracking.

The combined mass of the stylus, IRED, and vibration motor remained within the typical weight range of standard writing instruments (~23–25 g total), thereby minimizing alterations to natural reaching kinematics. All participants used the identical stylus configuration to ensure consistency across conditions.

The stylus displacement was recorded in three dimensions at 300 Hz using an Optotrak 3D Investigator motion capture system (Northern Digital Inc., Waterloo, Canada). The reaching surface was a 22-inch Dell touchscreen monitor (model E2222H) positioned flat on a table (approximately 76 cm height) in front of the participant. The task was presented via custom software design in E-Prime 3.0 (Psychology Software Tools, Sharpsburg, PA, USA), which also controlled the initiation of each trial, the presentation and timing of the visual stimuli, as well as augmented auditory and vibrotactile feedback.

Visual Stimuli: consisted of 2.5 × 2.5 cm yellow targets (square shape) presented on the touchscreen. The squares were arranged in three vertical pairs aligned with the participant’s midline. Within each pair, the two targets were spaced 35 mm apart horizontally (center-to-center), while adjacent pairs were separated vertically by 150 mm. These spatial parameters were selected based on established one-target advantage (OTA) paradigms (Lawrence et al., 2016; Khan et al., 2010), which have demonstrated that comparable inter-target distances reliably elicit sequential planning costs while maintaining task feasibility and accuracy. A designated “home” position (start square) was also displayed on the touchscreen for each hand at the beginning of each trial.

Augmented Feedback: The study included three sensory feedback conditions: No Feedback (NF): No additional sensory cues were given beyond intrinsic visual information from the screen. Auditory Feedback (AF): A brief 200 ms, 1,000 Hz tone (via a small piezoelectric buzzer) was presented as augmented auditory feedback. In our task, sensory feedback was provided only upon successful contact with the first target on the touchscreen. For auditory feedback (AF), a brief tone was delivered immediately when the stylus touched the first target in both one and two-target trials. Vibrotactile feedback (VF) was delivered via a small brushless DC vibration motor attached to the stylus, producing a 200 ms vibration upon hitting the first target. This augmented feedback offered immediate auditory or somatosensory information confirming that the first movement segment had been successfully completed. Both the auditory and vibrotactile feedback devices, as well as the initiation of the Optotrak recording, were controlled via the E-Prime interface through a Chronos response box, ensuring precise timing (the cues were triggered at the exact moment the stylus contacted the first target).

The decision to deliver augmented feedback exclusively at first-target contact was theoretically motivated. The One-Target Advantage (OTA) reflects the interaction between execution of the first movement segment and preparation of a subsequent action, making the first target a critical transition point between segments. Providing feedback at this juncture enabled us to examine whether enhanced sensory feedback confirming the first-segment completion influenced inter-segment planning processes. Importantly, Pause Time (PT) defined as the dwell time at Target 1 before initiation of the second movement, served as a key theoretical marker in distinguishing between the Movement Integration Hypothesis (MIH) and the Movement Constraint Hypothesis (MCH). Under the MIH, planning of the second segment is assumed to overlap with execution of the first segment, predicting shorter pause durations and a more continuous transition. In contrast, the MCH proposes that the first movement is strategically constrained to optimize the second movement, potentially resulting in longer stabilization at Target 1 before initiating the subsequent segment. Therefore, restricting feedback to the completion of the first movement segment allowed us to specifically evaluate how augmented sensory information interacts with this transition phase, without confounding ongoing online control processes.

### 2.3 Procedure

Participants completed five target-sequence conditions (task types), each crossed with three feedback conditions, resulting in 15 Task-Feedback blocks. Each block comprised 15 trials per Task-Feedback combinations. With the five task orientations performed under all three sensory conditions, there was a total of 225 trials completed.

The order of these blocks was counterbalanced across participants using a Latin Square design to avoid sequence effects. Participants were informed at the start of each block which task type and feedback condition they were about to perform, and they were given a short rest (~5 min) between blocks to minimize fatigue.

Prior to beginning the experimental trials participants performed a sensory screening task. The screening trials were completed to ensure participants could perceive the sensory feedback. They performed five trials of each feedback type (auditory and vibrotactile) in a randomized “go- no go” design. Participants were instructed to lift the stylus off the screen as soon as they detected the feedback stimulus, confirming that the cues were noticeable and timely.

**Figure 1:**
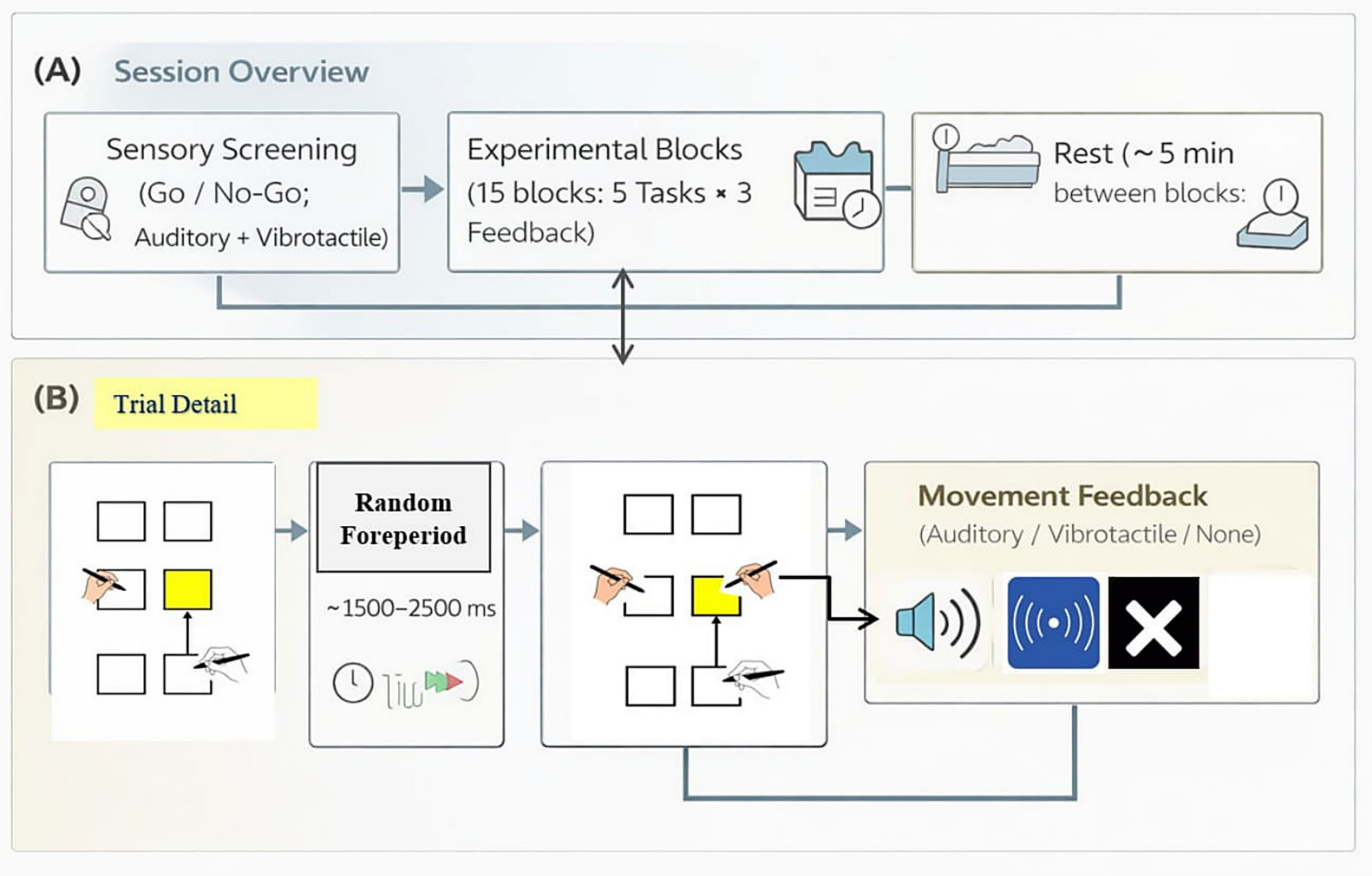
Experimental session overview and trial structure. **(A)** Session overview including sensory screening, experimental blocks, and rest periods. **(B)** Trial sequence illustrating the start position, randomized foreperiod (1500–2,500 ms), target presentation (go-signal), and movement feedback conditions (auditory, vibrotactile, or no feedback).

As shown in Figure 1, for all experimental trials, participants began by placing the styli on specified start positions and waiting for a go-signal: The right-hand stylus was placed on the right-side home square (start position for the right hand). And the left hand also had an initial position. Both hands were always placed on home positions at the start of every trial to ensure identical initial posture and biomechanical configuration across all task conditions, including bimanual reaching tasks. This standardized starting configuration ensured that any differences between unimanual and bimanual conditions were not attributable to differences in initial limb positioning. After an initial hold period on the start position, a randomized foreperiod (between 1,500–2,500 ms) elapsed, after which the go-signal was given. The go-signal consisted of the target square(s) on the screen changing from an outline to opaque, indicating that the participant should initiate their movement(s). The target opacity changed at the start of the trial (following the randomized foreperiod) and before any hand movement was initiated. The change served solely as the visual go-signal and did not provide information about the movement sequence. Participants were informed of the required task type (e.g., 1 T, 2T1He, 2T1Hr, 2T2He, 2T2Hr) at the beginning of each block, and the sequence remained constant throughout that block. Thus, participants knew in advance which reaching sequence to execute and the opacity change indicated when to begin the pre-specified movement.

**Figure 2:**
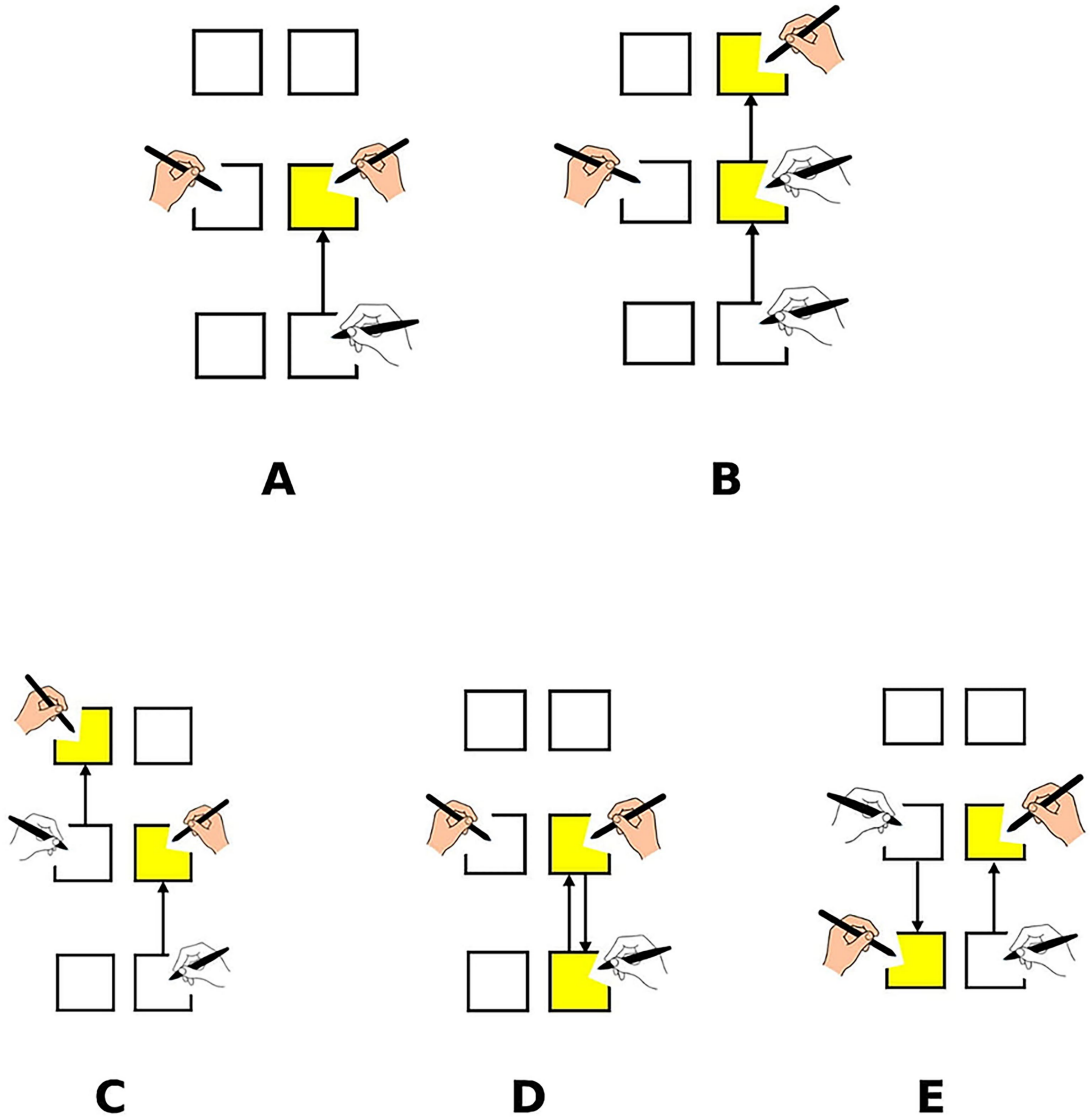
Schematic illustration of the five reaching task conditions. **(A)** One-target task (1 T). **(B)** Two-target single-hand extension (2T1He). **(C)** Two-target two-hand extension (2T2He). **(D)** Two-target single-hand reversal (2T1Hr). **(E)** Two-target two-hand reversal (2T2Hr). Yellow squares indicate target locations, and arrows represent movement direction.

Five types of reaching tasks were used as illustration in Figure 2: 1 T (One-Target, single movement): Participants used the right-hand stylus to move from the home position to Target 1 as quickly and accurately as possible. The left hand remained stationary on its designated start position. This condition represented an isolated, single aiming movement. 2T1He (Two-Target, One Hand, Extension): A two-segment sequence performed exclusively with the right hand. The movement began from the start position to Target 1, then continued outward from Target 1 to Target 2, located further along the same direction. The left hand remained stationary on Target 1 throughout the trial. 2T1Hr (Two-Target, One Hand, Reversal): A two-segment sequence with the right hand but involving a reversal of direction. The right hand first moved to Target 1, then reversed course back toward the start position (or a nearby location) for the second segment. The left hand again remained stationary on Target 1. 2T2He (Two-Target, Two Hands, Extension): A bimanual sequence in which the right hand moved first from its start position to Target 1. Following this, the left hand executed the second movement, extending forward from its resting position on left Target 1 to left Target 2. Each hand contributed one movement in sequence. 2T2Hr (Two-Target, Two Hands, Reversal): A bimanual sequence similar to 2T2He, except that the left hand performed a reversal movement. After the right hand reached Target 1, the left hand moved from left Target 1 back toward its home position (a proximal return movement).

Throughout all trials, participants were instructed to move “as quickly and accurately as possible” to the target(s) in response to the go-signal, and to make the movements in one smooth motion per segment (though normal corrective movements were permitted).

### 2.4 Dependent measures

We calculated a range of temporal and spatial kinematic measures for each trial, separately for the first movement segment (from start to Target 1) and the second movement segment (Target 1 to Target 2 or reversal/return target, when applicable). Precise computational algorithms for each dependent variable are provided in Table 1.

**Table 1:** Dependent variables, and computational algorithms.

Category	Variable	Computational definition/algorithm
Temporal measures	RT1	Time from go-signal onset to movement onset, where movement onset is the first sample with resultant velocity > 30 mm/s for ≥30 ms.
Temporal measures	MT1	Time from movement onset to Segment 1 end, where Segment 1 end is defined as the first sample after Target 1 acquisition where resultant velocity < 30 mm/s for ≥30 ms
Temporal measures	PT	Time from Segment 1 end (velocity < 30 mm/s for ≥30 ms at Target 1) to Segment 2 onset (first sample with velocity > 30 mm/s for ≥30 ms following Segment 1 end).
Temporal measures	MT2	Time from Segment 2 onset to Segment 2 end, where Segment 2 end is the first sample where resultant velocity < 30 mm/s for ≥30 ms at the final target.
Kinematic measures	PV1	Maximum resultant velocity (mm/s) during Segment 1 (from movement onset to Segment 1 end).
Kinematic measures	TTPV1	Time from movement onset to the sample at which PV1 occurs (within Segment 1).
Kinematic measures	TAPV1	Time from the PV1 sample to Segment 1 end (deceleration duration in Segment 1).
Kinematic measures	PV2	Maximum resultant velocity (mm/s) during Segment 2 (from Segment 2 onset to Segment 2 end).
Kinematic measures	TTPV2	Time from Segment 2 onset to the sample at which PV2 occurs (within Segment 2).
Kinematic measures	TAPV2	Time from the PV2 sample to Segment 2 end (deceleration duration in Segment 2).
Spatial accuracy measures	CE1	Signed endpoint error at Target 1, computed as the signed distance (mm) between endpoint position at Segment 1 end and Target 1 center, where sign is defined along the primary movement axis.
Spatial accuracy measures	VE1	Standard deviation (mm) of CE1 across trials within each participant × condition.
Spatial accuracy measures	CE2	Signed endpoint error at final target, computed as the signed distance (mm) between endpoint position at Segment 2 end and final target center, sign defined as above.
Spatial accuracy measures	VE2	Standard deviation (mm) of CE2 across trials within each participant × condition.

Position data were low-pass filtered using a dual-pass second-order Butterworth filter (cutoff = 15 Hz). Movement onset and offset were determined using a velocity threshold of 30 mm/s sustained for at least 30 ms.

### 2.5 Data processing and analysis

The raw positional data from the Optotrak was first filtered using a dual-pass second-order Butterworth filter with a cutoff frequency of 15 Hz. This low-pass filtering smoothed the trajectory while preserving the key motion characteristics. The cutoff frequency of 15 Hz for the dual-pass second-order Butterworth filter was selected based on established conventions in goal-directed reaching research using Optotrak systems sampled at 200–300 Hz. Human reaching movements typically contain the majority of their signal energy below 10–15 Hz; therefore, a 15 Hz cutoff effectively attenuates high-frequency measurement noise while preserving genuine movement dynamics. Pilot inspection of power spectra from representative trials confirmed that movement-related frequency content was concentrated below this range. Movement onset and offset were defined using the velocity threshold of 30 mm/s sustained for at least 30 ms as this threshold-based method is widely used in kinematic analyses of aiming movements to prevent false detections due to tremor, minor postural adjustments, or measurement noise. The requirement that velocity remain above (or below) the threshold for 30 ms ensured stable onset detection. Visual inspection of randomly selected trials confirmed that this algorithm reliably captured true movement initiation and termination without truncating early acceleration phases or deceleration corrections.

Participants also completed an individualized calibration where they were asked to hold the stylus at the center of each target square for about 2 s. This provided reference data to map the recorded Optotrak coordinates to the participant’s perceived target centre on the touchscreen, allowing us to compute the spatial errors (CE and VE) accurately.

First, trials in which participants made an obvious error (e.g., initiating too early, missing the target entirely, or any technical tracking errors) were excluded from further analysis. Next, outliers were identified and removed. We defined outliers quantitatively as trials where any key metric main dependent variables, RT, MT, PVs fell more than ±2.5 standard deviations from the participant’s mean for that condition. Approximately 5% of trials were flagged as outliers and removed the remaining trials were used for statistical analysis.

The Optotrak motion capture system provides sub-millimeter spatial resolution and high temporal precision, making it well suited for capturing fine-grained kinematic variables in reaching paradigms. The use of standardized velocity thresholds for movement onset and offset detection, combined with low-pass filtering and individualized calibration procedures, ensured consistent and reliable extraction of temporal and spatial measures across participants. Similar preprocessing pipelines have been widely adopted in goal-directed reaching research, supporting the validity of the derived kinematic measures. The 30 mm/s velocity threshold sustained for 30 ms minimized false-positive onset detection due to tremor or noise, ensuring robust identification of movement initiation.

We used repeated-measures Analysis of Variance (ANOVA) to analyze the effects of Feedback condition and Task type on the various dependent measures. For measures associated with the first segment (RT1, MT1, PV1, etc.), a 3 × 5 ANOVA was performed: Feedback Condition (NF vs. Auditory vs. Vibrotactile) × Target Task (1 T, 2T1He, 2T1Hr, 2T2He, 2T2Hr), with all factors within-subjects. For second-segment measures (which only exist for the two-target tasks), we used a 3 × 4 ANOVA: Feedback (3 levels) × Target Task (only the four two-target tasks). Where Mauchly’s test indicated violations of sphericity, we adjusted degrees of freedom using the Greenhouse–Geisser correction. The normality of residuals was assessed for each dependent variable using Shapiro–Wilk tests. Significant main effects or interactions were followed up with Bonferroni-adjusted pairwise comparisons to identify specific differences between conditions. An alpha level of 0.05 was used for determining statistical significance. Effect sizes for ANOVA (partial eta squared, ηp^2^) are reported to indicate the magnitude of effects.

All statistical analyses were conducted in SPSS (version 28.0; IBM Corp., Armonk, NY, USA). The data were organized so that each participant contributed mean values for each combination of feedback and task (after trial-level filtering) in the ANOVAs.

#### 2.5.1 Study reporting standards

This study is classified as an experimental study using a within-subject repeated-measures design. In accordance with recommendations from the EQUATOR Network, the TREND reporting guideline was consulted as the most appropriate framework for reporting experimental intervention studies. A completed reporting checklist is provided in Supplementary material 1.

## 3 Results

Full descriptive statistics and ANOVA tables are provided in Supplementary material 2.

### 3.1 Temporal measures

#### 3.1.1 Reaction time

Asignificant main effect of Sensory Condition was found for reaction time, *F*_2, 46_ = 6.67, *p* = 0.003, η_p_^2^ = 0.23. *Post hoc* comparisons indicated faster RTs in the no feedback condition (M = 294 ms, SE = 7 ms) compared to auditory [M = 303 ms, SE = 8 ms; ΔM = −9.10 ms, 95% CI (−17.47, −0.73), *p* = 0.03] and vibrotactile feedback [M = 310 ms, SE = 11 ms; ΔM = −16.10 ms, 95% CI (−30.20, −2.00), *p* = 0.02]. No significant difference was found between auditory and vibrotactile feedback [ΔM = −6.99 ms, 95% CI (−18.04, 4.05), *p* = 0.35].

Moreover, a significant main effect of Target Task was also observed, *F*_4, 92_ = 6.94, *p* < 0.001, η_p_^2^ = 0.23. ΔM = 17.24 ms, 95% CI [3.01, 31.46], *p* = 0.01. RTs were significantly slower in the two-target two-hand reversal task (2T2Hr; M = 326 ms, SE = 11 ms) compared to 2T1He, ΔM = 42.84 ms, 95% CI [15.76, 69.93], *p* = 0.001, and compared to 2T1Hr, ΔM = 32.39 ms, 95% CI [4.53, 60.24], *p* = 0.015. No other pairwise differences were significant. The Sensory Condition × Target Task interaction was not significant, *F*_8, 184_ = 1.63, *p* = 0.12, η_p_^2^ = 0.07.

**Figure 3:**
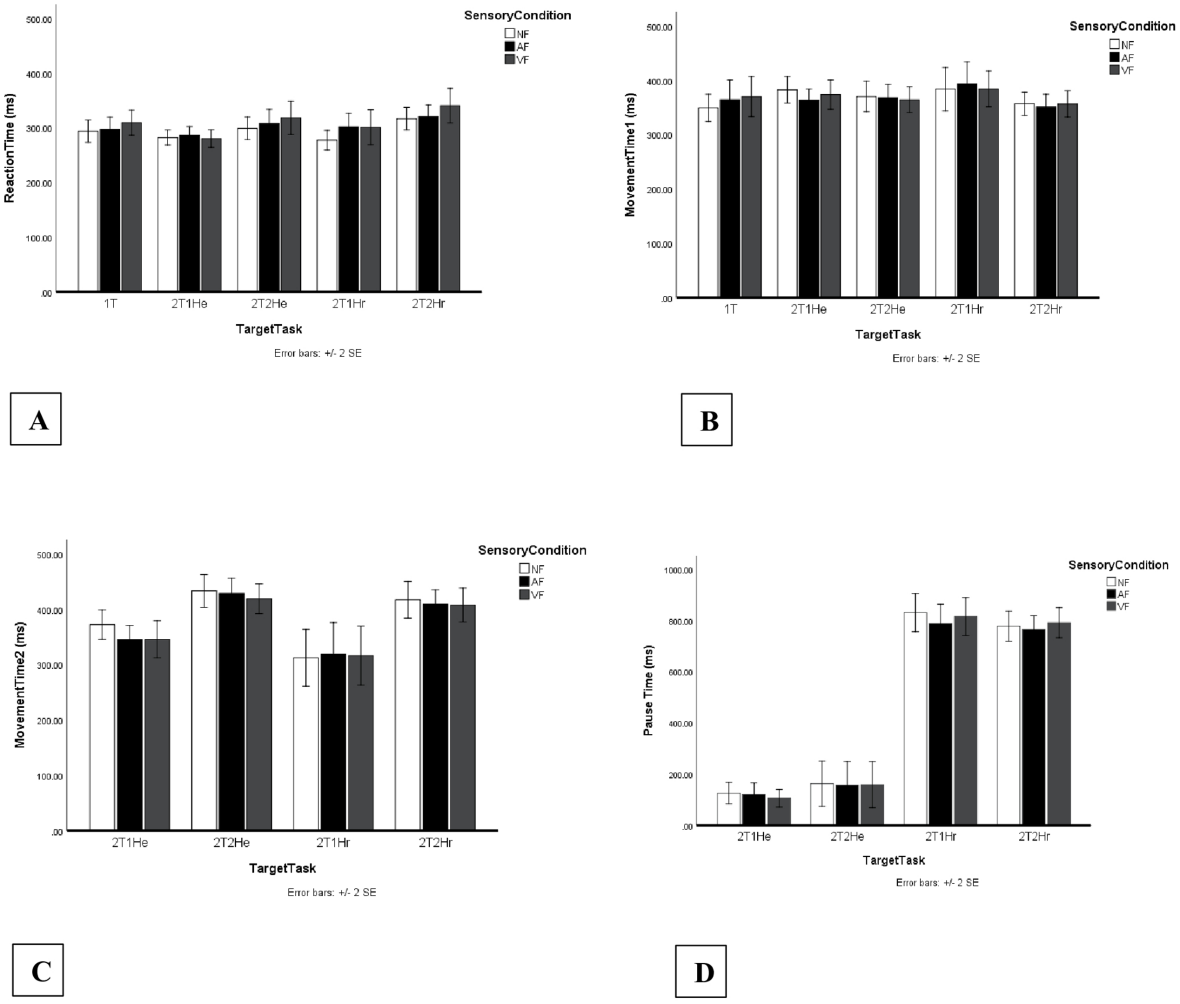
Temporal dependent variables (ms) across task and sensory conditions. Bars represent mean values across no-feedback (NF), auditory feedback (AF), and vibrotactile feedback (VF) conditions. Segment 1 measures are presented across five task configurations including one one-target task (1T) and four two-target tasks (2T1He, 2T2He, 2T1Hr, 2T2Hr). Segment 2 measures are presented across four two-target tasks (2T1He, 2T2He, 2T1Hr, 2T2Hr). Error bars indicate standard errors. Panels display: **(A)** Reaction time (RT, ms), **(B)** Movement time segment 1 (MT1, ms), **(C)** Movement time segment 2 (MT2, ms), and **(D)** Pause time (ms).

Figure 3A displays the mean reaction times for all Sensory Condition × Target Task combinations.

#### 3.1.2 Movement time to the first target (MT1)

A repeated-measures ANOVA revealed no significant main effect of Sensory Condition on MT1, *F*_2, 46_ = 0.09, *p* = 0.911, η_p_^2^ = 0.004. Similarly, there was no significant main effect of Target Task, *F*_4, 92_ = 1.63, *p* = 0.174, η_p_^2^ = 0.07. The interaction between Sensory Condition and Target Task was also not significant, *F*_8, 184_ = 1.73, *p* = 0.093, η_p_^2^ = 0.07.

The distribution of mean MT1 values across Sensory Condition × Target Task combinations is shown in Figure 3B.

#### 3.1.3 Movement time to the second target (MT2)

The main effect of Sensory Condition on MT2 was not significant, *F*_2, 46_ = 1.80, *p* = 0.177, η_p_^2^ = 0.07. A significant main effect of Target Task was observed, *F*_3, 69_ = 17.67, *p* < 0.001, η_p_^2^ = 0.43. *Post hoc* Bonferroni comparisons revealed that MT2 was significantly longer in the two-target two-hand extension task (2T2He; M = 427 ms, SE = 13 ms) compared to the two-target single-hand extension task (2T1He; M = 355 ms, SE = 12 ms), ΔM = 72.45 ms, 95% CI [45.27, 99.63], *p* < 0.001, and compared to the two-target two-hand reversal task (2T2Hr; M = 412 ms, SE = 14 ms), ΔM = 56.83 ms, 95% CI [20.22, 93.44], *p* = 0.001. Furthermore, MT2 was significantly shorter in the two-target single-hand reversal task (2T1Hr; M = 316 ms, SE = 26 ms) compared to 2T2He, ΔM = 111.13 ms, 95% CI [49.30, 172.95], *p* < 0.001, and compared to 2T2Hr, ΔM = 95.51 ms, 95% CI [32.18, 158.85], *p* = 0.001.

The Sensory Condition × Target Task interaction was not significant, *F*_6, 138_ = 0.92, *p* = 0.480, η_p_^2^ = 0.04.

Figure 3C presents the mean MT2 values for each Sensory Condition × Target Task combination.

#### 3.1.4 Pause time (PT)

A significant main effect of target task type was found on pause time, *F*_3, 69_ = 192.61, *p* < 0.001, η_p_^2^ = 0.89, indicating differences in pause duration across the four movement tasks. Post-hoc comparisons revealed that the two-handed conditions 2T2He and 2T2Hr resulted in significantly longer pause times compared to the one-handed tasks, 2T1He and 2T1Hr. No significant difference emerged between 2T2He and 2T2Hr, suggesting that the nature of the second movement (extension vs. reversal) does not substantially influence pause duration in two-handed movements. Similarly, 2T1He and 2T1Hr showed comparable pause times, indicating that one-handed movements are executed with minimal pause regardless of the direction of the second movement. There was no main effect of sensory feedback condition, nor was there a significant interaction between target task and sensory feedback type.

Figure 3D displays the mean pause times across all Sensory Condition × Target Task combinations.

### 3.2 Spatial accuracy

#### 3.2.1 Constant error at the second target (CE2)

No significant main effect of Sensory Condition was found, *F*_2, 46_ = 0.74, *p* = 0.481, η_p_^2^ = 0.03.

A significant main effect of Target Task was found, *F*(3, 69) = 7.90, p < 0.001, ηp^2^ = 0.26. *Post hoc* Bonferroni comparisons revealed that constant error was significantly more negative in the two-target single-hand extension task (2T1He; M = −0.81, SE = 0.26) compared to the two-target two-hand extension task (2T2He; M = 0.87, SE = 0.35), ΔM = −1.68, 95% CI [−2.94, −0.43], *p* = 0.005, and compared to the two-target two-hand reversal task (2T2Hr; M = 0.65, SE = 0.39), ΔM = −1.46, 95% CI [−2.79, −0.14], *p* = 0.025. Additionally, constant error was significantly greater in 2T2He compared to the two-target single-hand reversal task (2T1Hr; M = −0.33, SE = 0.34), ΔM = 1.21, 95% CI [0.02, 2.39], *p* = 0.044.

The interaction between Sensory Condition and Target Task approached significance, *F*_6, 138_ = 2.1, *p* = 0.055, η_p_^2^ = 0.08.

**Figure 4:**
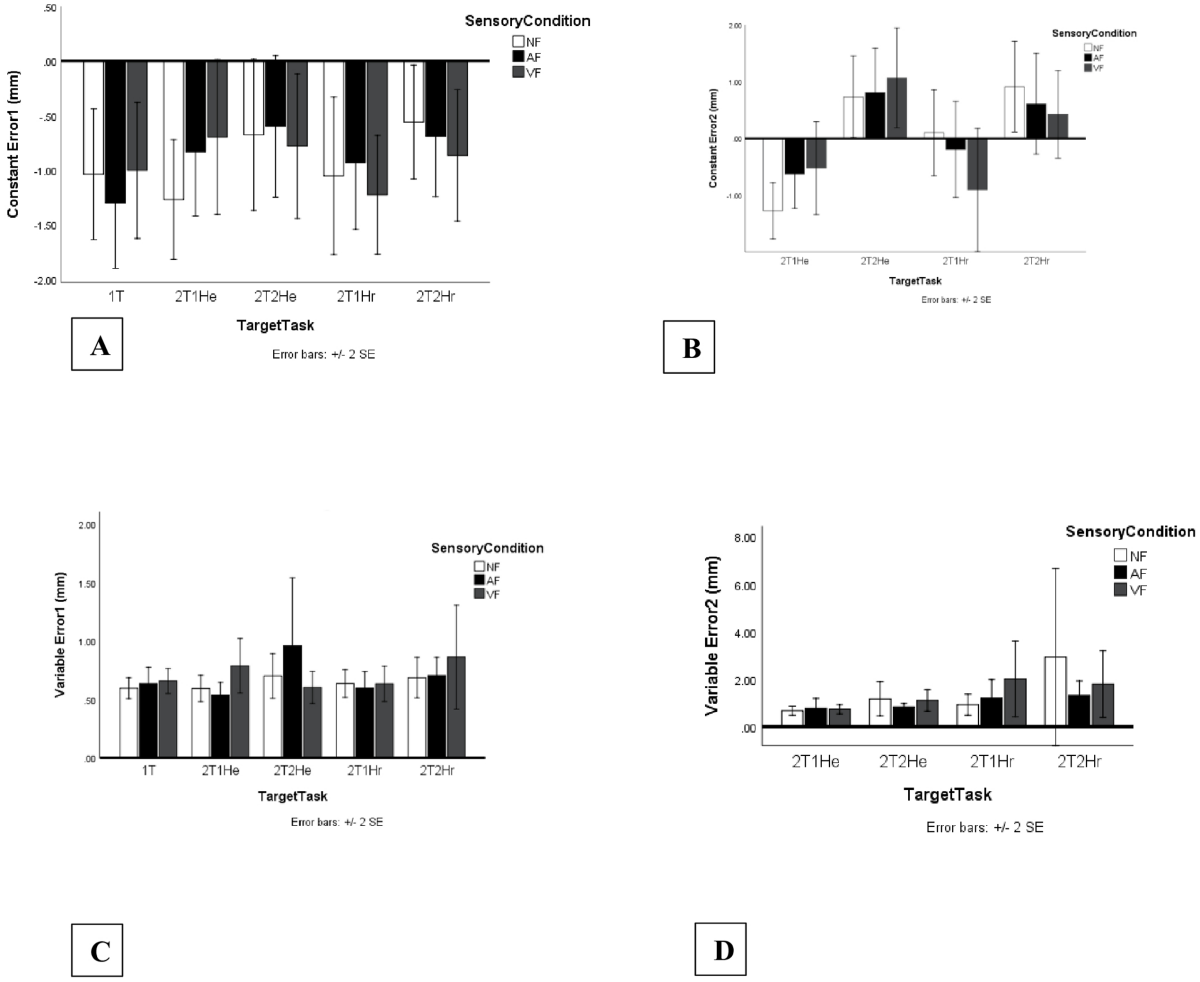
Spatial accuracy measures (mm) across sensory feedback and task conditions. Bars represent mean spatial accuracy outcomes across no-feedback (NF), auditory feedback (AF), and vibrotactile feedback (VF) conditions. Segment 1 measures are presented across five task configurations (1T, 2T1He, 2T2He, 2T1Hr, 2T2Hr), while segment 2 measures are presented across four two-target tasks (2T1He, 2T2He, 2T1Hr, 2T2Hr). Error bars indicate standard errors. Panels include: **(A)** Constant error segment 1 (CE1, mm), **(B)** Constant error segment 2 (CE2, mm), **(C)** Variable error segment 1 (VE1, mm), and **(D)** Variable error segment 2 (VE2, mm). Positive constant error values reflect overshooting, whereas negative values indicate undershooting relative to the target location.

Figure 4 displays the mean CE2 values for all Sensory Condition × Target Task combinations (NF, AF, VF across 1 T, 2T1He, 2T2He, 2T1Hr, 2T2Hr), illustrating the overall distribution of directional error across task configurations. Non-significant spatial accuracy outcomes (CE1, VE1, and VE2) are also presented in Figure 4 for completeness.

### 3.3 Kinematic control

#### 3.3.1 Peak velocity during the first movement segment (PV1)

No significant main effect of Sensory Condition was found, *F*_2, 46_ = 1.08, *p* = 0.350, η_p_^2^ = 0.04. A significant main effect of Target Task was observed, *F*_4, 92_ = 3.57, *p* = 0.009, η_p_^2^ = 0.13. Peak velocity was significantly higher in the one-target task (M = 969 mm/s, SE = 36 mm/s) compared to the two-target single-hand extension task (2T1He; M = 898 mm/s, SE = 29 mm/s), ΔM = 70.54 mm/s, 95% CI [6.66, 134.43], *p* = 0.023.

**Figure 5:**
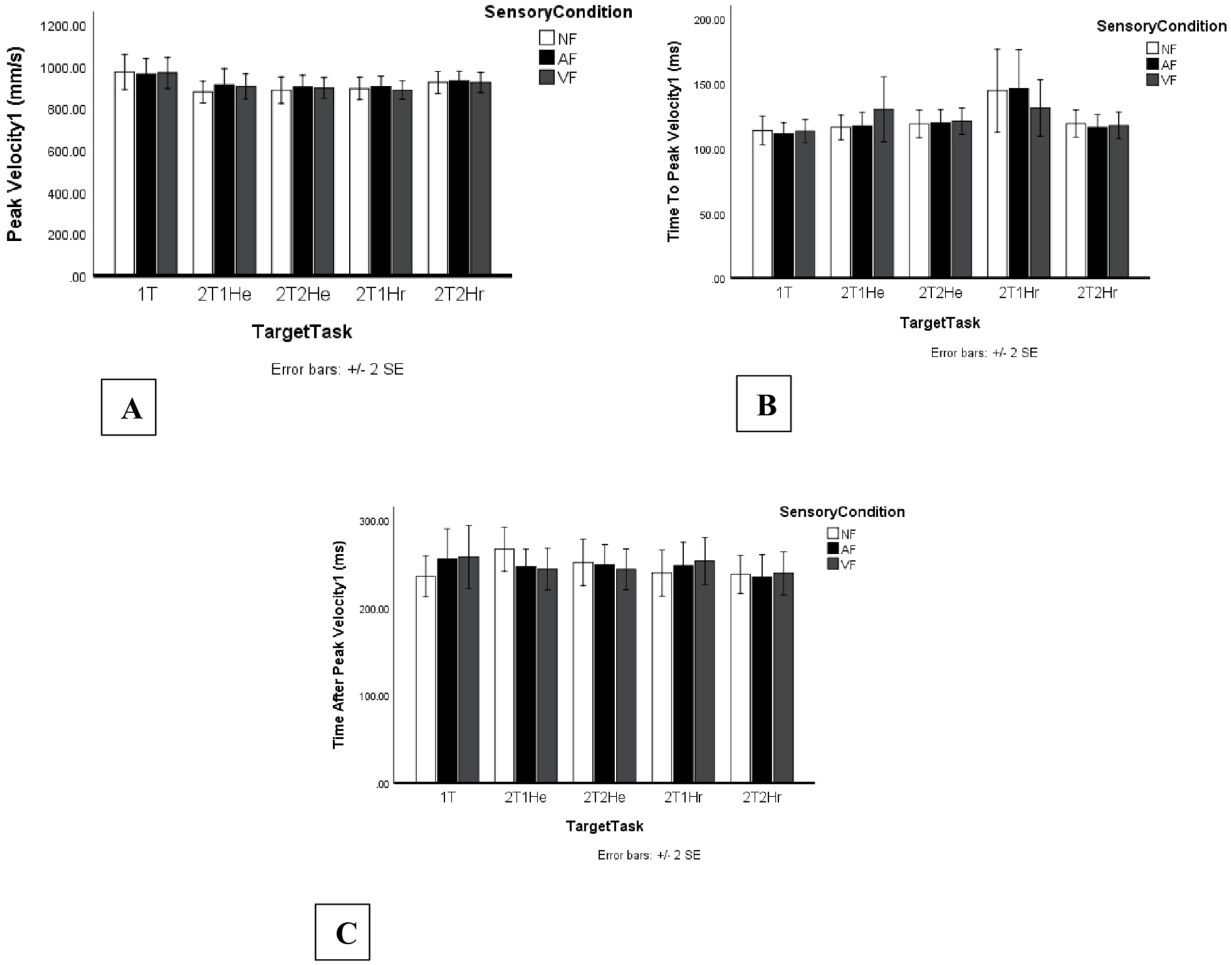
Kinematic dependent variables for movement segment 1 across sensory feedback and task conditions. Bars represent mean kinematic outcomes across no-feedback (NF), auditory feedback (AF), and vibrotactile feedback (VF) conditions for five task configurations, including one one-target task (1T) and four two-target tasks (2T1He, 2T2He, 2T1Hr, 2T2Hr). Error bars indicate standard errors. Panels include: **(A)** Peak velocity segment 1 (PV1, mm/s), **(B)** Time to peak velocity segment 1 (TTPV1, ms), and **(C)** Time after peak velocity segment 1 (TAPV1, ms).

Figure 5 presents the mean kinematic values for all Sensory Condition × Target Task combinations, including (a) PV1, (b) TTPV1, and (c) TAPV1.

#### 3.3.2 Time to peak velocity (TTPV2)

**Figure 6:**
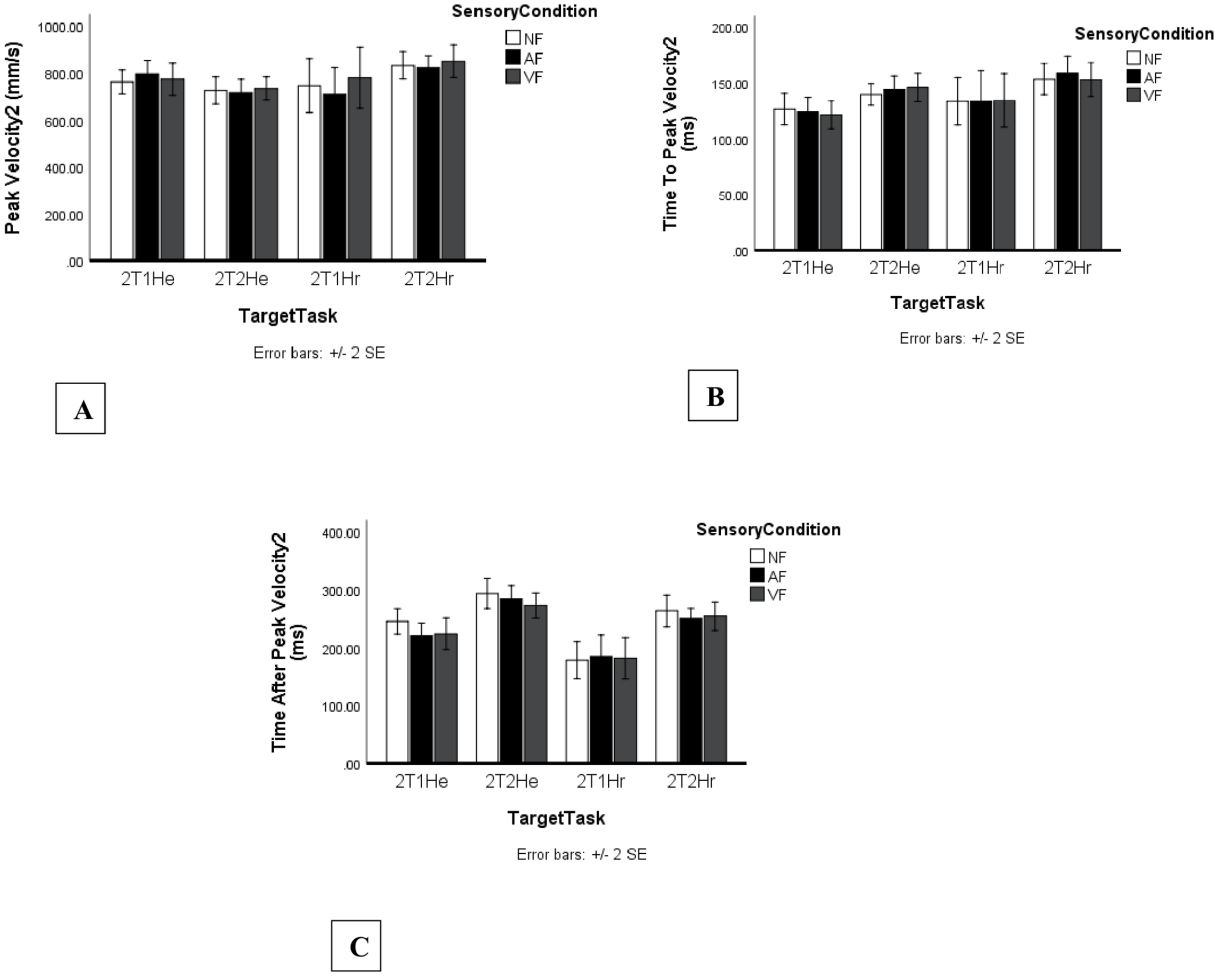
Kinematic dependent variables for movement segment 2 across sensory feedback and task conditions. Bars represent mean kinematic outcomes across no-feedback (NF), auditory feedback (AF), and vibrotactile feedback (VF) conditions for four two-target task configurations (2T1He, 2T2He, 2T1Hr, 2T2Hr). Error bars indicate standard errors. Panels include: **(A)** Peak velocity segment 2 (PV2, mm/s), **(B)** Time to peak velocity segment 2 (TTPV2, ms), and **(C)** Time after peak velocity segment 2 (TAPV2, ms).

However, for TTPV2, a significant main effect of Target Task was observed, *F*_3, 69_ = 4.82, *p* = 0.004, η_p_^2^ = 0.173. *Post hoc* Bonferroni comparisons indicated that participants reached peak velocity significantly earlier in the two-target single-hand extension task (2T1He; M = 124 ms, SE = 6 ms) compared to the two-target two-hand extension task (2T2He; M = 143 ms, SE = 5 ms), ΔM = −19.13 ms, 95% CI [−34.45, −3.81], p = 0.009, and compared to the two-target two-hand reversal task (2T2Hr; M = 155 ms, SE = 7 ms), ΔM = −31.02 ms, 95% CI [−50.71, −11.34], *p* = 0.001. Figure 6 presents the complete pattern of second-segment kinematic means across Sensory Condition × Target Task combinations.

#### 3.3.3 Time after peak velocity (TAPV2)

The main effect of Sensory Condition was statistically significant. *F*_2, 46_ = 4.23, *p* = 0.020, η_p_^2^ = 0.156. As shown in Figure 6, vibrotactile feedback resulted in faster post-peak movement times (M = 230 ms, SE = 11 ms) compared to no feedback (M = 246 ms, SE = 10 ms) (ΔM = 12.01 ms, 95% CI [−0.04, 24.05], *p* = 0.05).

A significant main effect of Target Task was also observed, *F*_3, 69_ = 27.18, *p* < 0.001, η_p_^2^ = 0.54.

Post hoc Bonferroni comparisons indicated that TAPV2 was significantly longer in the two-target two-hand extension task (2T2He; M = 284 ms, SE = 11 ms) compared to the two-target single-hand extension task (2T1He; M = 231 ms, SE = 10 ms), ΔM = 53.32 ms, 95% CI [28.98, 77.66], p < 0.001, and compared to the two-target single-hand reversal task (2T1Hr; M = 182 ms, SE = 17 ms), ΔM = 101.82 ms, 95% CI [62.27, 141.38], *p* < 0.001. Additionally, TAPV2 was significantly longer in the two-target two-hand reversal task (2T2Hr; M = 256 ms, SE = 11 ms) compared to 2T1Hr, ΔM = 74.31 ms, 95% CI [35.46, 113.16], p < 0.001. Furthermore, TAPV2 was significantly longer in 2T1He compared to 2T1Hr, ΔM = 48.50 ms, 95% CI [8.99, 88.01], *p* = 0.010.

## 4 Discussion

This study investigated how augmented sensory feedback, specifically auditory and vibrotactile stimulation, impact the planning and execution of sequential reaching movements in young adults. We were particularly interested in whether such feedback could modulate the OTA. The results provide several insights into both theoretical and practical aspects of sequential motor control.

Under intrinsic conditions (i.e., no augmented feedback), the expected one-target advantage (OTA) was not consistently observed. Contrary to the classic finding of longer reaction times for sequences (Henry and Rogers, 1960; Adam et al., 2000), we found that the simple two-target extension with one hand (2T1He) did not elicit a longer reaction compared to a single-target task. In fact, participants initiated that two-target movement just as fast, or even faster, than the one-target movement. This is an intriguing result because it suggests that when the two-target sequence is very predictable and perhaps viewed as a “natural” continuation (here, an outward extension), young adults might not experience the typical planning delay. Our findings align with recent observations from our lab’s work with older adults, where providing vibrotactile feedback actually led to faster reaction times in two-target tasks than one-target tasks (effectively reversing the traditional OTA in reaction time) (Kolahdouz et al., 2025; Mohammadalinezhad Kolahdouz et al., 2026).

In the present study without feedback, younger adults may have effectively “pre-planned” the extension sequence so well that there was little extra cost at movement initiation or possibly even an anticipatory priming effect knowing that an ongoing movement was required. It is worth noting that the more complex sequences (especially 2T2Hr) still showed longer RTs, reinforcing that complexity does add planning time. But the fact that 2T1He did not incur an RT penalty suggests that the one-target advantage in RT is not universal; it can be mitigated under favorable conditions (e.g., a single effector, continuous direction). This nuance contributes to evidence that the OTA is context-sensitive (Lavrysen et al., 2002). In short, younger adults can sometimes plan a two-step action almost as readily as a one-step action, challenging the notion that simply having a second segment invariably delays the start. An unexpected finding was the main effect of Sensory Condition on reaction time, despite augmented feedback being delivered only at target contact. Because the feedback occurred after movement onset, it could not have directly influenced online motor execution at the movement initiation. However, participants were aware of the feedback condition at the beginning of each block. This blocked design may have induced differences in the preparatory state, expectancy, or attentional allocation. Anticipating sensory feedback has been shown to engage motor preparatory mechanisms even before feedback is delivered, influencing neural responses in motor and sensory systems and potentially modulating reaction times. For example, anticipation of virtual feedback in a P300-BCI task elicited preparatory sensorimotor activity prior to movement, indicating that expected feedback can influence brain activity before the sensory event itself (Syrov et al., 2023). In addition, recent work on anticipatory planning shows that when individuals expect particular movement demands or sensory consequences, motor planning processes are engaged ahead of movement execution, influencing reaction times and preparatory activity (Zhang et al., 2024).

In this context, the absence of feedback may have reduced cognitive load or sensory expectancy, leading to faster movement initiation, consistent with resource-sharing accounts of preparatory and planning processes and with the idea that attentional focus and expectancy can influence motor performance (Wulf and Lewthwaite, 2016). Conversely, anticipating auditory or vibrotactile stimulation may have introduced a modest preparatory cost, slightly prolonging reaction time. Thus, the RT differences likely reflect contextual or strategic adjustments at the planning stage rather than direct sensory-motor processing effects.

Movement Execution and Task Complexity: Although the first movement time (MT1) was similar across tasks, the second movement time (MT2) clearly reflected the influence of task complexity. Consistent with previous research (e.g., the movement constraint idea and our prior findings with older adults), the second segment took the longest to execute in the most complex scenario (two hands with extension) and was fastest in the simplest task (same hand reversal). The single-hand reversal (2T1Hr) was performed particularly quickly, likely because reversing direction to a nearby point is mechanically easier (the hand’s momentum can be quickly redirected back) (Lawrence et al., 2016). The bimanual extension (2T2He) was slowest, which fits with the idea that when a new effector and a continued outward goal are involved, people proceed more cautiously, or simply need more time to accomplish the task. This pattern aligns with the movement constraint hypothesis as participants appeared to execute the first movement more cautiously when a demanding second movement was forthcoming, resulting in longer total sequence times. Indeed, in our study, second-movement accuracy (CE2) was highest in the simplest tasks and worst in the hardest tasks, reinforcing the idea that higher task demands reduces overall movement precision (Lavrysen et al., 2002).

The fact that bimanual sequences had larger endpoint errors and longer times suggests additional coordination costs possibly because coordinating two hands (even sequentially) is more complex both from a behavioral and neural perspective. That is, the “receiving” hand might not be as prepared for its movement as when the same hand continues. Our findings are in line with prior multi-limb control studies, which have noted that introducing either an extra limb or a more difficult second movement can reduce accuracy and increase time. Shea et al. (2016) showed that coordinating discrete, out-of-phase movements across limbs such as during bimanual tasks with differing timing or amplitude increases coordination difficulty and prolongs movement execution. In our study, although MT1 alone did not differ significantly, MT2 increased with task complexity, reinforcing that movement demands grow as sequences become more complex (Shea et al., 2016). Movement strategy adjustments observed in the present study can also be interpreted through a dual-task or central-capacity lens. In sequential aiming, performers initiate and execute the first segment while simultaneously preparing the second, which can create competition for shared processing resources during response selection and action programming. Such interference aligns with classic capacity-sharing models showing that performance deteriorates when concurrent action demands compete for limited central resources (Wu et al., 2024; Tolotti et al., 2025). More recent integrative work similarly emphasizes that multitasking costs often reflect constraints at central stages of action selection and cognitive control, particularly when tasks require re-programming, effector switching, or increased coordination demands (Strobach, 2024). In our more complex conditions especially 2T2He and 2T2Hr recruiting a different effector and/or reversing direction likely increased central load, contributing to longer RTs, prolonged pauses at Target 1, and longer MT2. This perspective complements movement integration and movement constraint accounts by highlighting how attentional and central processing limits can amplify sequencing costs when task complexity increases. In terms of the kinematic factors, participants adjusted their movement strategies according to task demands and sensory feedback, as evidenced by their velocity profiles. In the one-target condition, peak velocity during the first segment (PV1) was significantly higher than in the 2T1He (two-target, same-hand extension) task. This finding supports the notion that single-target movements are executed through a rapid, pre-planned control strategy, likely governed by a ballistic motor command with minimal reliance on online correction (Khan et al., 2010).

In contrast, sequential tasks particularly 2T1He, 2T2He (two-hand extension), and 2T2Hr (two-hand reversal) were characterized by lower PV1 values, reflecting a more conservative velocity profile. These results align with the movement constraint hypothesis, which posits that when sequential accuracy demands are present, performers strategically modulate speed and control in anticipation of upcoming target transitions (Khan et al., 2010; Lawrence et al., 2013).

Further supporting this interpretation, Time to Peak Velocity in the second segment (TTPV2) was significantly longer in the 2T2He condition compared to both 2T1He and 2T2Hr. This suggests that greater task complexity, such as switching hands combined with outward extension, delays the attainment of peak movement speed. Within the framework of the Multiple Process Model of goal-directed movement (Elliott et al., 2010, 2017), this pattern reflects an increased reliance on online control and sensory processing when executing more complex sequential actions.

Although TTPV2 increased with complexity, PV2 did not show significant differences across sensory conditions; any numerical increases under vibrotactile feedback were not statistically reliable.

However, a significant main effect of Sensory Condition was observed for Time After Peak Velocity in the second movement segment (TAPV2). Vibrotactile feedback produced lower mean TAPV2 values than no feedback, indicating a tendency toward faster post-peak movement completion and more efficient late-phase control. At the same time, TAPV2 was significantly affected by Target Task, with the longest durations observed in the more complex two-hand conditions, especially 2T2He. Together, these findings suggest that the deceleration phase of sequential reaching is sensitive to both sensory context and movement complexity. This interpretation is broadly consistent with previous work showing that vibrotactile feedback can support terminal movement adjustments and improve end-phase control (Raveh et al., 2018). As mentioned above MT2 did not differ significantly between sensory feedback conditions. Although vibrotactile feedback did not significantly change MT2, PV2, or TTPV2, it did shorten the deceleration phase (TAPV2), suggesting that feedback supported more efficient online control specifically during the end stage of the movement. However, MT2 did vary significantly across task types: 2T2He had the longest MT2, while 2T1Hr (same-hand reversal) was the shortest. These results reinforce that task complexity directly affects movement duration, consistent with earlier findings showing increased demands in multi-limb or directionally complex movements (Shea et al., 2016).

A nuanced effect of vibrotactile feedback was observed in TAPV1 during the 2T1Hr task. Vibrotactile feedback led to slightly longer time after peak velocity in the first segment. According to the Multiple Process Model, this can be interpreted as a shift toward limb-target control, where participants use sensory confirmation to ensure target contact before reversing direction (Elliott et al., 2010, 2017). Rather than a performance cost, this prolonged TAPV1 reflects a strategic adjustment to enhance accuracy under conditions that require rapid reprogramming and motor redirection.

Despite this temporary increase in TAPV1, the second movement in 2T1Hr remained the fastest (lowest MT2), indicating that the feedback-supported adjustment at the first target ultimately benefited the fluidity of the sequence. This is consistent with Sigrist et al. (2013), who observed that augmented feedback can prompt brief slowdowns to incorporate new information sources, especially when spatial precision is critical.

Finally, the efficiency of reversal movements in 2T1Hr may also reflect mechanical advantages. Reversing direction toward a proximal target likely requires less spatial recalibration, as momentum can be redirected more easily supporting interpretations offered by Lawrence et al. (2013) regarding reversal mechanics.

Several limitations should be considered when interpreting the present findings. First, the sample size was modest. Although sufficient to detect medium-to-large effects, larger samples would increase statistical precision and enhance confidence in the robustness of the observed interaction patterns. Second, the experimental paradigm was conducted in a highly controlled laboratory setting. While this design allowed for precise measurement of kinematic variables and isolation of sequential planning effects, it may not fully reflect the complexity and variability of real-world motor sequence performance. Everyday motor behavior typically involves dynamic environmental demands and less predictable movement structures. Third, although participants completed multiple repetitions across conditions, potential learning or strategy adaptation across blocks was not formally modeled. The blocked design may have allowed participants to refine anticipatory strategies over time. Future studies would benefit from examining trial-by-trial adaptation or incorporating randomized designs to better isolate practice effects. Finally, the present study focused exclusively on young neurotypical adults. Therefore, the conclusions are specific to this population and should not be generalized to other age groups or clinical populations without direct empirical evidence. Future research examining older adults or individuals with neuromotor impairments will help determine whether the observed sensory-feedback effects extend beyond healthy young participants.

## 5 Conclusion

In summary, this study demonstrates that augmented vibrotactile feedback can positively influence the execution of sequential aiming movements, particularly by enhancing the efficiency of the latter phase of movement (reducing the deceleration time) and aiding the quick initiation of subsequent actions. The classical one-target advantage, which has long been viewed as a fixed cost of sequencing movements, is not immune to such intervention we observed contexts in which it was minimized or absent (even without feedback, in a highly practiced extension sequence, and with vibrotactile feedback in more complex sequences). Our findings support a view of sequential movement control as a flexible process where the motor system can allocate resources to either plan ahead (integration) or play it safe (constraint) providing additional sensory information, such as vibrotactile stimulation, directly influences this allocation by promoting greater reliance on online control mechanisms. Specifically, tactile feedback appears to encourage a mode of control that allows for both speed and accuracy: participants did not overshoot or become erratic (accuracy was maintained or improved), yet they were able to move faster through the critical phases of the task.

A future goal of this research is to optimize motor performance in both healthy individuals and those with motor impairments. Going forward, integrating such sensory feedback in real-world settings: sports, surgical training, rehabilitation for stroke or age-related motor decline could prove beneficial. It is important to note, however, that task complexity itself had a greater overall influence on motor outcomes than sensory feedback alone. This supports the idea that sensory augmentation might be especially useful in situations where task demands place heavier load on the motor control system. Beyond its theoretical contributions, the present findings have meaningful practical implications for health professionals, particularly occupational therapists and rehabilitation specialists. Sequential motor tasks are central to daily activities such as dressing, eating, writing, tool manipulation, and object transfer. Our findings suggest that vibrotactile feedback may enhance the efficiency of movement execution, particularly during the deceleration phase of sequential actions. Shorter post-peak velocity times indicate improved terminal control, which is critical for accurate object placement and fine motor adjustments. In clinical settings, therapists often aim to improve both speed and precision of goal-directed movements without increasing compensatory strategies. The present results suggest that integrating simple vibrotactile cues at key task events (e.g., target contact confirmation) may support more efficient online control without compromising accuracy. This approach could be particularly beneficial in rehabilitation contexts where individuals demonstrate slowed movement termination or impaired sensory integration. Moreover, the observation that task complexity significantly influences motor planning and execution underscores the importance of graded task design in therapy. Therapists may strategically manipulate movement complexity (e.g., same-limb vs. alternating-limb sequences) to progressively challenge motor planning systems while monitoring cognitive load. Vibrotactile augmentation may serve as a supportive tool during higher-demand tasks.

Although this study examined healthy young adults, the mechanisms identified here may inform future applications in populations with sensorimotor deficits, including stroke survivors, older adults, or individuals with neurological conditions, where optimizing sequential motor performance is essential for functional independence.

## Data Availability

Raw data supporting the conclusions of this article are available from the authors upon request.
