# Supplementary material for: Vibrotactile augmentation enhances late-phase control in sequential reaching without accuracy costs
Source: Front Hum Neurosci. 2026 Apr 24;20:1794200. doi: 10.3389/fnhum.2026.1794200 (PMC13153064; doi:10.3389/fnhum.2026.1794200)
Supplement: Supplementary file 1 [file Supplementary_File_1.docx]

| **Section** | **Item** | **Location in manuscript** |
| --- | --- | --- |
| Title | Study identified as experimental study | Title page |
| Abstract | Structured summary of background, purpose, methods, results | Abstract |
| Background | Scientific rationale for sensory feedback in motor control | Introduction |
| Objectives | Research aims and hypotheses | Introduction |
| Study design | Within-subject repeated-measures experimental design | Methods |
| Participants | Inclusion and exclusion criteria | Methods – Participants |
| Sample size | Number of participants included in analysis | Methods – Participants |
| Experimental task | Description of one-target and two-target movements | Methods – Task |
| Apparatus | Motion tracking system, stylus, vibrotactile device | Methods – Apparatus |
| Intervention | Vibrotactile and auditory feedback conditions | Methods – Sensory conditions |
| Procedure | Sequence of trials and task instructions | Methods – Procedure |
| Outcome measures | RT, MT, PV, TTPV, TAPV, CE, VE | Methods – Dependent variables |
| Data processing | Movement onset criteria and filtering procedures | Methods – Data processing |
| Statistical analysis | repeated-measures ANOVA | Methods – Statistical analysis |
| Results | Descriptive and inferential statistics | Results |
| Interpretation | Discussion of findings related to sensory feedback | Discussion |
| Ethics approval | Institutional ethics approval and consent | Methods – Participants |
| Data availability | data sharing statement | End of manuscript |
