# Supplementary material for: Vibrotactile augmentation enhances late-phase control in sequential reaching without accuracy costs
Source: Front Hum Neurosci. 2026 Apr 24;20:1794200. doi: 10.3389/fnhum.2026.1794200 (PMC13153064; doi:10.3389/fnhum.2026.1794200)
Supplement: Supplementary file 2 [file Supplementary_File_2.docx]

Supplementary Material

**Supplementary Table 1s.**

Summary of temporal outcome measures. Main effects of Sensory Condition, Target Task, and their interactions are reported for reaction time (RT), first-segment movement time (MT1), second-segment movement time (MT2), and pause time (PT). F-statistics, p-values, and partial eta squared (ηp²) are shown. Asterisks (*) denote statistically significant effects (*p* ≤ .05).

| **Source** | **RT** | **MT1** | **MT2** | **PT** |
| --- | --- | --- | --- | --- |
| **Sensory Condition** | F(2,46)=6.67, p=.003*, η_p_²=.23 | F(2,46)=0.09, p=.911, η_p_²=.004 | F(2,46)=1.80, p=.177, η_p_²=.07 | F(2,46)=.685, p=.509, η_p_²=.029 |
| **Target Task** | F(4,92)=6.94, p<.001*, η_p_²=.23 | F(4,92)=1.63, p=.174, η_p_²=.06 | F(3,69)=17.67, p<.001*, η_p_²=.43 | F(3,69) =192.61, p<.001*, η_p_²=.89 |
| **Sensory × Task** | F(8,184)=1.63, p=.120, η_p_²=.07 | F(8,184)=1.73, p=.093, η_p_²=.07 | F(6,138)=0.923, p=.480, η_p_²=.04 | F(6,138)=.386, p=.887, η_p_²=.016 |

**Supplementary Table 2s.**

Summary of spatial accuracy measures. Main effects of Sensory Condition, Target Task, and their interaction are reported for constant error at the first (CE1) and second (CE2) targets, as well as variable error at the first (VE1) and second (VE2) targets. F-statistics, p-values, and partial eta squared (ηp²) are provided. Asterisks (*) indicate statistically significant effects (*p* ≤ .05).

| **Source** | **CE1** | **VE1** | **CE2** | **VE2** |
| --- | --- | --- | --- | --- |
| **Sensory Condition** | F(2,46)=0.194, p=.825, η_p_²=.008 | F(2,46)=4.73, p=.626, η_p_²=.020 | F(2,46)=0.74, p=.481, η_p_²=.031 | F(2,46)=.462, p=.633, η_p_²=.020 |
| **Target Task** | F(4,92)=1.524, p=.202, η_p_²=.062 | F(4,92)=.864, p=.489, η_p_²=.036 | F(3,69)=7.90, p<.001*, η_p_²=.256 | F(3,69)=2.187, p=.097, η_p_²=.087 |
| **Sensory × Task** | F(8,184)=1.713, p=.098, η_p_²=.069 | F(8,184)=1.14, p=.343, η_p_²=.047 | F(6,138)=2.11, p=.055, η_p_²=.084 | F(6,138)=.639, p=.698, η_p_²=.027 |

**Supplementary Table 3s.**

Summary of kinematic control measures. Main effects of Sensory Condition, Target Task, and their interaction are reported for peak velocity at the first (PV1) and second (PV2) movement segments, time to peak velocity at the first (TTPV1) and second (TTPV2) segments, and time after peak velocity at the first (TAPV1) and second (TAPV2) segments. F-statistics, p-values, and partial eta squared (η_p_²) are presented. Asterisks (*) indicate statistically significant effects (*p* ≤ .05).

| **Source** | **PV1** | **PV2** | **TTPV1** | **TTPV2** | **TAPV1** | **TAPV2** |
| --- | --- | --- | --- | --- | --- | --- |
| **Sensory Condition** | F(2,46)=1.08, p=.350, η_p_²=.05 | F(2,46)=1.60, p=.212, η_p_²=.065 | F(2,46)=0.012, p=.98, η_p_²=.001 | F(2,46)=2.36, p=.791, η_p_²=.010 | F(2,46)=0.070, p=.932, η_p_²=.003 | F(2,46)=4.23, p=.020*, η_p_²=.156 |
| **Target Task** | F(4,92)=3.57, p=.009*, η_p_²=.13 | F(3,69)=1.83, p=.149, η_p_²=.074 | F(4,92)=3.368, p=.061, η_p_²=.128 | F(3,69)=4.822, p=.004*, η_p_²=.173 | F(4,92)=.634, p=.639, η_p_²=.027 | F(3,69)=27.176, p<.001*, η_p_²=.54 |
| **Sensory × Task** | F(8,184)=4.77, p=.872, η_p_²=.020 | F(6,138)=1.905, p=.084, η_p_²=.076 | F(8,184)=1.43, p=.183, η_p_²=.059 | F(6,138)=.578, p=.748, η_p_²=.025 | F(8,184)=1.439 p=.183, η_p_²=.0.059 | F(6,138)=1.315, p=.254, η_p_²=.054 |
